# Preoperative Narcotic Education in Spine Surgery: A Retrospective Study

**DOI:** 10.3390/jcm13226644

**Published:** 2024-11-06

**Authors:** Anas M. Abbas, Alex Ngan, Jian H. Li, Araf M. Abbas, Aadi Pandya, Salman Ahmad, Bongseok Jung, Shaya Shahsavarani, Rohit B. Verma

**Affiliations:** Department of Orthopedic Surgery, Northwell Health, New Hyde Park, NY 11040, USA

**Keywords:** education, narcotics, opioid crisis, orthopedic surgery, spine, spine surgery

## Abstract

**Background/Objectives**: The objective of this study was to determine whether preoperative opioid education reduces opioid consumption after spine surgery and which educational methods are the most effective. Orthopedists are the most likely to prescribe opioids among all specialists. To alleviate the prescription opioid crisis, studies have identified ways to taper narcotic dosage and use following surgery. The role of preoperative education and its varying modalities on opioid consumption following spine surgery has yet to be reported in the literature. **Methods**: The study group received formal education describing the use of opioids, side effects, and alternatives to pain management. Patients were to choose their preferred modality of a 2 min narrated video and two handouts to be watched and read in their individual time, attending a small class led by a physician assistant where they watched the 2 min narrated video along with reading the two handouts or receiving a one-on-one session with the treating spine surgeon. Meanwhile, the control group received standard preoperative education. Refill prescriptions were collected from patients’ electronic medical record charts at the 2-week, 1-month, 3-month, and 6-month postoperative follow-ups. The primary outcome measure was morphine equivalents (MME) of prescription opioids at six months following spine surgery. **Results**: At 2 weeks postoperatively, there were no statistically significant differences between patients who received any formal narcotic education and those who did not. At 1 and 3 months postoperatively, the video education group (*p*-value < 0.001), class education group (*p*-value < 0.001), and the one-on-one education group (*p*-value < 0.05) all had significant reductions in opioid consumption. At 6 months postoperatively, only the video education group (*p*-value < 0.001) and the class education group (*p*-value < 0.01) had significant reductions in opioid consumption. **Conclusions**: A two-fold approach with a video and handouts significantly decreases the prescription dosage at six months postoperatively and allows for early opioid cessation after undergoing spine surgery.

## 1. Introduction

The aging population of the United States (US) poses a challenge in combating the opioid crisis. Furthermore, one in five Americans suffers from chronic pain, most commonly back pain [1]. Guidelines for treating chronic back pain recommend opioids as next-line treatment for patients who are unresponsive to non-steroidal inflammatory drugs (NSAIDs) [2]. Patients prescribed opioids for diseases of the musculoskeletal system had increased odds of chronic opioid prescription usage [3]. In initial visits with complaints of low back pain, 21.6% of patients were prescribed opioids [4].

Orthopedists are the most likely to prescribe opioids among all specialists [5]. Currently, treatment modalities for back pain include NSAIDs, physical therapy, epidural steroid injections, and, as a last resort, surgery. Unfortunately, the first 3 days after surgery are often accompanied with severe surgical site pain, prompting spine surgeons to prescribe opioids [6]. After lumbar spine surgery, it has been found that two-thirds of patients become long-term opioid users (defined as >90 days [7]), and 8.4% of patients met the criteria for chronic opioid use the second year after surgery [8].

To alleviate the prescription opioid crisis, studies have identified ways to taper narcotic dosage and use following surgery. The 2018 Neers Award for the American Shoulder and Elbow Surgeons (ASES) demonstrated a significant reduction in postoperative narcotic consumption after preoperative narcotic education in patients who underwent arthroscopic rotator cuff repair [9]. The low health literacy of the detriments and adverse effects of narcotics may contribute to chronic opioid use after orthopedic surgery. Consequently, preoperative narcotic education before spine surgery may be a leading modality in the earlier cessation of opioids, mitigating a spine surgeon’s role in the opioid epidemic.

The role of preoperative education and its varying modalities on opioid consumption following spine surgery has yet to be reported in the literature. The purpose of this study was to determine whether preoperative opioid education reduces opioid consumption after spine surgery and which educational methods are the most effective. Our study group had the option to choose from three different educational modalities: a 2 min narrative video with two handouts to be watched and read at any time prior to surgery, a physician assistant-led class that also included the same video and handouts, or a one-on-one session with the treating surgeon. This is the first study that looked at the effects of preoperative narcotic education in spine surgery and the first to analyze the most effective modality of education delivery. We hope this simple intervention can decrease orthopedists’, specifically spine surgeons’, role in the US opioid crisis.

## 2. Materials and Methods

### 2.1. Inclusion and Exclusion Criteria

Eligible patients were found through Northwell Health Orthopedic Institute’s registry of spine surgery. This study included patients who underwent spine surgery at North Shore University Hospital from January 2021 to December 2021 and January 2022 to December 2022. Patients with a history of gastroesophageal reflux disease, allergies, or sensitivity to prescription opioids were excluded. Operations were performed by the fellowship-trained orthopedic spine surgeon senior author.

The data collected included preoperative demographics such as age during surgery, sex, race, insurance, and body mass index (BMI). Preoperative prescription opioid usage and comorbid conditions (specifically high blood pressure, diabetes, anxiety or depression, and current alcohol and tobacco use) were also collected. Patients undergoing fusions, decompressions, laminectomies, discectomies, and anterior cervical decompression and fusion (ACDF) of 3 levels or fewer were analyzed in this study.

The study group were eligible patients who underwent spine surgery from 1 January 2022 to 31 December 2022. The study group received formal education describing the use of opioids, side effects, tolerance, dependence, addiction, and alternatives to pain management. Patients were to choose their preferred modality of a 2 min narrated video (File S1) and two handouts (Figures S1 and S2) to be watched and read in their leisure time, attending a small class led by a physician assistant where they watched the 2 min narrated video along with reading the two handouts, or receiving a 30 min to 1 h one-on-one session with the treating spine surgeon that consisted of standard preoperative education including indications for surgery, surgical technique, and expected postoperative recovery and pain management. The treating surgeon led postoperative pain management discussions by reading the handouts to ensure that there was no variability in the patient’s understanding of the use, detriments, and alternatives of postoperative narcotics. Meanwhile, the control group received standard preoperative education and underwent surgery from 1 January 2021 to 31 December 2021.

### 2.2. Outcome Measures

Refill prescriptions were collected from patients’ electronic medical record charts at the 2-week, 1-month, 3-month, and 6-month postoperative follow-ups. The primary outcome measure was morphine equivalents (MME) of prescription opioids at six months following spine surgery.

### 2.3. Statistical Analysis

Linear regression models were developed to estimate the effects of demographic factors, comorbid factors (high blood pressure, diabetes, anxiety or depression, current alcohol use, and current tobacco use), and the type of education received on MME at 2 weeks, 1 month, 3 months, and 6 months postoperatively. All statistical analyses were completed in R version 4.3.2 (Boston, MA, USA).

## 3. Results

Of the 222 control group patients who underwent spine surgery between January 2021 and December 2021, 150 met the inclusion criteria. Patients were excluded for meeting the exclusion criteria (n = 66) or being lost to follow-up (n = 6). Of the 217 study group patients who underwent spine surgery between January 2022 and December 2022, 137 met the inclusion criteria. Patients were excluded for meeting the exclusion criteria (n = 78) or being lost to follow-up (n = 2). Patient demographics are listed in Table 1.

**Figure 1:**
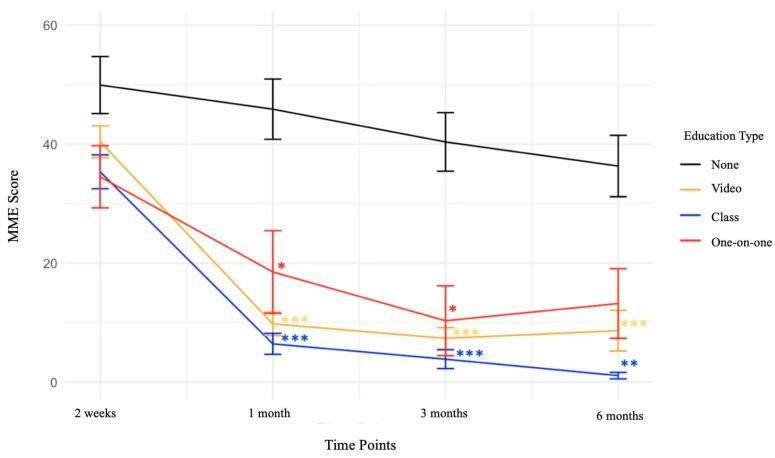
Line graph of MME scores for each type of education with standard error bars. *p*-value labels: * = <0.05, ** = <0.01, *** = <0.001. MME, morphine milligram equivalent.

At 2 weeks postoperatively, there were no statistically significant differences between patients who received any formal narcotic education and those who did not (Figure 1).

At 1 month postoperatively, patients who received video education had a significant reduction in the MME of prescription opioids, with an average reduction of 32.40 compared to patients undergoing normal preoperative education (*p*-value < 0.001). Patients who received class education also had significant reductions in the MME of prescription opioids, with an average reduction of 32.58 compared to patients who did not receive any formal narcotic education (*p*-value < 0.001). Patients who received one-on-one education also had a statistically significant reduction in MME, averaging a 22.60-point reduction (*p*-value < 0.05) (Table 2, Figure 1).

At 3 months postoperatively, both patients receiving video education and those receiving class education had a significant reduction in the MME of prescription opioids, with average reductions of 28.48 and 31.90, respectively, compared to patients undergoing normal preoperative education (*p*-value < 0.001). Patients receiving one-on-one education also had a statistically significant reduction in MME at 3 months postoperatively, with an average reduction of 23.16 (*p*-value < 0.05) (Table 2, Figure 1).

At 6 months postoperatively, patients who received video education had a significant reduction in the MME of prescription opioids, with an average reduction of 26.58 compared to patients undergoing normal preoperative education (*p*-value < 0.001). Patients who received class education also had significant reductions in the MME of prescription opioids, with an average reduction of 28.26 compared to patients who did not receive any formal narcotic education (*p*-value < 0.01). Patients who received one-on-one education did not have statistically significant reductions in MME at 6 months postoperatively (Table 2, Figure 1).

## 4. Discussion

The purpose of this study was to determine whether preoperative narcotic education would decrease opioid use in MME and promote early cessation after undergoing spine surgery. Our interventions included preoperative narcotic education via patients’ preferred modality of a 2 min narrated video and the two handouts to be watched and read prior to surgery in private, attending a physician assistant-led class to watch the 2 min narrated video and read the two handouts, or receiving a one-on-one session between the treating surgeon and the patient. To our knowledge, this is the first orthopedic study to compare the efficacy of various educational modalities in reducing narcotic consumption and the first study to analyze the effects of preoperative narcotic education on spine surgery patients.

We found a significant decrease in narcotic consumption in the 1-month and 3-month follow-ups after surgery in both the video-, in-person class-, and one-on-one-educated groups. At the 6-month follow-up, the video- and class-educated groups continued to have a reduced MME usage compared to those that did not receive formal education. One-on-one sessions between the surgeon and patient did not yield any significant reduction at the 6-month time point. We attribute this result to the decreased effectiveness of verbal education as an instructional tool and likely a limited amount of time for preoperative opioid counseling given a surgeon’s high clinical responsibilities. Another possible theory is the lack of standardization in one-on-one teaching as compared to the more regimented video and class education. A recent study on preoperative opioid education in orthopedics reinforces our findings revealing that studies that employ a two-fold approach to delivering education through either a handout and in-personal educational session or a handout and video yield superior outcomes compared to those involving only verbal counseling [10]. Another study on patients undergoing various surgical interventions determined that opioid-specific video-based education significantly reduced postoperative opioid consumption and pain score but had no influence on refill request, opioid leftovers, or opioid use after hospital discharge [11].

Multiple studies have shown that preoperative narcotic usage affects postoperative opioid consumption. Our multivariate analysis found that preoperative narcotic usage was a significant factor predicting the final postoperative narcotic consumption at 1, 3, and 6 months. Interestingly, those that smoked and consumed alcohol had a statistically significant increase in MME usage at 6 months, but smoking and alcohol alone were not significant 6-month MME predictors. The interaction effect between smoking and alcohol had a greater effect size in MME than prior MME history at 6 months. Patients with a history of alcohol abuse were observed to have a lower pain threshold, indicating that they had a higher sensitivity to pain [12]. In addition, there is a significant association between the frequency of alcohol consumption and an increase in opioid consumption [13]. The low pain threshold followed by severe postoperative pain may explain the increase in MME regardless of receiving preoperative narcotic education [12,13]. Another predisposing factor to a greater postoperative MME was tobacco smoking within one month before surgery [14]. Patients with a recent history of tobacco smoking had greater opioid use levels compared to patients who quit smoking more than 1 month before their operation [14].

At the 6-month follow-up in our study, both the video- and class-educated groups demonstrated a significant reduction in the MME score compared to the control group, while the one-on-one-educated group did not. Since this is still deemed a short-term follow-up, a long-term follow-up interval of >2 years of our study would provide more insight into the long-term benefits of opioid education. In a follow-up study for the 2018 Neers Award paper, patients preoperatively educated in opioid use who underwent ARCR continued to have decreased opioid dependency at 2 years [9].

The US is currently undergoing an opioid epidemic. The year 2020 was the worst year on record, with over 69,000 deaths attributed to opioid overdoses [15]. It is postulated that this crisis has been exacerbated by personal grief or trauma and the inability of many people with opioid use disorder to access in-person treatments centers that were shut down because of the COVID-19 pandemic [16,17]. It is estimated that nearly one-third of people who abuse drugs start with prescription opioid medicines [18]. Responding to this crisis is difficult and complex. Neurobiological vulnerabilities and social determinants of health are also variables to consider among individuals [19]. As orthopedists make up the highest percentage of medical specialists prescribing narcotics, it is vital that orthopedists investigate and apply effective strategies for combating this public health crisis.

Intense postoperative pain associated with spine surgeries can possibly be prevented with proper perioperative pain management [20]. Interventions, such as multimodal analgesic pain regimens, can be utilized in the postsurgical period to reduce postoperative opioid consumption [21,22]. An effective analgesic regimen to provide adequate postoperative pain management after undergoing complex spine surgery is using preoperative or intraoperative paracetamol and cyclooxygenase-2-specific inhibitors or NSAIDs, with opioids used postoperatively as a rescue analgesic [20]. A systematic review found that in patients undergoing various orthopedic surgical procedures with interventions including the use of local anesthetics and/or nerve blocks (42 studies), NSAIDs (31 studies), neuropathic pain medications (9 studies), and multimodal analgesic combinations (25 studies), 127 studies (90.1%) demonstrated a significant decrease in postoperative opioid consumption compared to the control interventional with a median reduction of 39.7% [23].

However, physician-controlled limitation in narcotic prescriptions without patient insight and input may negatively affect patient satisfaction and the recovery process. Thus, collaborating with and involving the patient in a shared decision framework through interactive preoperative education can yield numerous benefits. Another variable to consider is effective communication between the entire surgical team in terms of understanding physician preferences for opioid prescriptions. A study by Esposito et al. sought to explore proper opioid discharge guidelines and ways to improve adherence [24]. They discovered a significant difference between attending preferences and what residents and advanced practice providers (APPs) believed were the attending’s preferences [24]. A total of 11% of attendings preferred their patients to “most of the time” or “always” receive opioids on discharge, whereas up to 45% of residents and 54% of advanced practice providers shared similar sentiments [24]. Overall, 57% of attendings reported they “most of the time” or “always” communicated their discharge preferences, while 12% of residents communicated theirs [24]. This study demonstrated the importance of communication within the treating surgical team to prevent deviating from opioid guidelines and unnecessarily prescribing opioids postoperatively [24].

Our study highlights that something as easy and quick as a 2 min video and handouts can significantly reduce a patient’s narcotic consumption after spine surgery. Since there were no significant differences in our initial patient cohort between the control and study groups, preoperative narcotic education may help patients manage their expectations and anxiety about postoperative pain. As the perception of pain is multifactorial, it is also important for surgeons to appreciate the subtleties of pain as opposed to simply increasing narcotic prescriptions [25,26]. Studies have demonstrated preoperative narcotic education to be a relatively simple, readily accessible, and cost-effective strategy, so we anticipate that this intervention can be applied to all elective orthopedic procedures and even additional specialties [9,10,27,28]. Our study found that both the video education and in-class education groups demonstrated the greatest reductions in MME usage. The common denominator between the two groups was the utilization of the 2 min narrated video with associated handouts. The video and handouts focused on the risks and adverse events of opioid dependence along with viable alternative treatments for postoperative pain management. The in-class-educated group had the greatest reduction in MME scores. We believe that having a licensed medical professional nearby to address further questions or concerns was beneficial in the patients’ understanding of opioid consumption.

This study is not without limitations. Postoperative patients under a single spine surgeon at our hospital were analyzed. A surgeon’s preference for postoperative pain management protocol could influence the length and dosage of narcotics taken after surgery. A study including patients under multiple surgeons may provide different results. Furthermore, our prescription monitoring program only identifies filled prescriptions and is limited to identifying illicit use. To reduce this bias, we included subjects that were captured in a state central database. A limitation in this database was that we had prior knowledge of the dosage and date of prescription following the surgeries. However, we did not inquire about the frequency or quantity of pill consumption. In addition, patients with a history of legitimate opioid use versus opioid abuse were not determined in this study. Thus, in patients with reported prior opioid use, we could not determine whether this represents acute postoperative use or a history of opioid addiction. Furthermore, prescription history is only saved in the previous 12 months from the date of search. Patients who underwent surgery before the 12 months within our search history had their prescriptions recorded by searching their chart within the electronic medical record. Another limitation of this study is that it included a mixed cohort of less invasive spine surgeries in both the study group and control group. The surgeries included in this study were fusions, decompressions, laminectomies, discectomies, and ACDF of 3 levels or fewer. Instrumentation or the extent of construct or length for the types of surgeries included may lead to varying postoperative recovery periods and necessities of narcotic pain management. Lastly, patients in our study were allowed to choose their educational modality, leading to self-selection bias where patients could choose the intervention that they believe they would learn and retain the most from. Consequently, the effectiveness of our educational modalities may not be applicable to all post-surgical spine patients.

## 5. Conclusions

This retrospective chart review found that preoperative narcotic education via a two-fold approach with a video and handouts significantly decreased the prescription dosage at six months postoperatively and allowed for early opioid cessation after undergoing spine surgery. Such preoperative education may be an efficient, cost-effective way to reduce opioid use for future spine surgeries

## Figures and Tables

**Table 1:** Preoperative demographics.

Preoperative Demographics	
Variable	N = 287
Age, yr	60.84 ± 14.1
Male sex	165 (57.4%)
BMI, kg/m^2^	29.1 ± 6.3
Smoking	101 (35.1%)
Alcohol	116 (40.4%)
High Blood Pressure	130 (45.2%)
Diabetes	66 (22.9%)
Anxiety/Depression	23 (8.0%)
Prior MME Usage	120 (41.8%)
Race	
White	152 (52.9%)
Black	47 (16.3%)
Asian	25 (8.7%)
Latino	27 (9.4%)
Other	36 (12.5%)

N, number; yr, year; BMI, body mass index; kg/m^2^, kilogram/meters^2^; MME, morphine milligram equivalent.

**Table 2:** Regression results: effects of different types of education relative to no education on MME score.

Postoperative Time	Video Education	*p*-Value	Class Education	*p*-Value	One-on-One Education	*p*-Value
2 Weeks	−6.41	0.382	−11.51	0.165	−11.42	0.273
1 Month	−32.4	<0.001	−32.58	<0.001	−22.6	0.035
3 Months	−28.48	<0.001	−31.9	<0.001	−23.16	0.024
6 Months	−26.58	<0.001	−28.26	<0.01	−17.4	0.111

Significance was set to *p*-value < 0.05.

## Data Availability

The original contributions presented in the study are included in the article, further inquiries can be directed to the corresponding authors.

## Supplementary Materials

The following supporting information can be downloaded at https://www.mdpi.com/article/10.3390/jcm13226644/s1, Figure S1: Pain management infographic; Figure S2: Pain management bullet list; File S1: Informative pain management video.
