# Supplementary material for: Preoperative Narcotic Education in Spine Surgery: A Retrospective Study
Source: J Clin Med. 2024 Nov 6;13(22):6644. doi: 10.3390/jcm13226644 (PMC11594543; doi:10.3390/jcm13226644)
Supplement: Supplementary file 1 [file jcm-13-06644-s001.zip › Opioid Study Final-090721 2.pptx]

## Slide 1
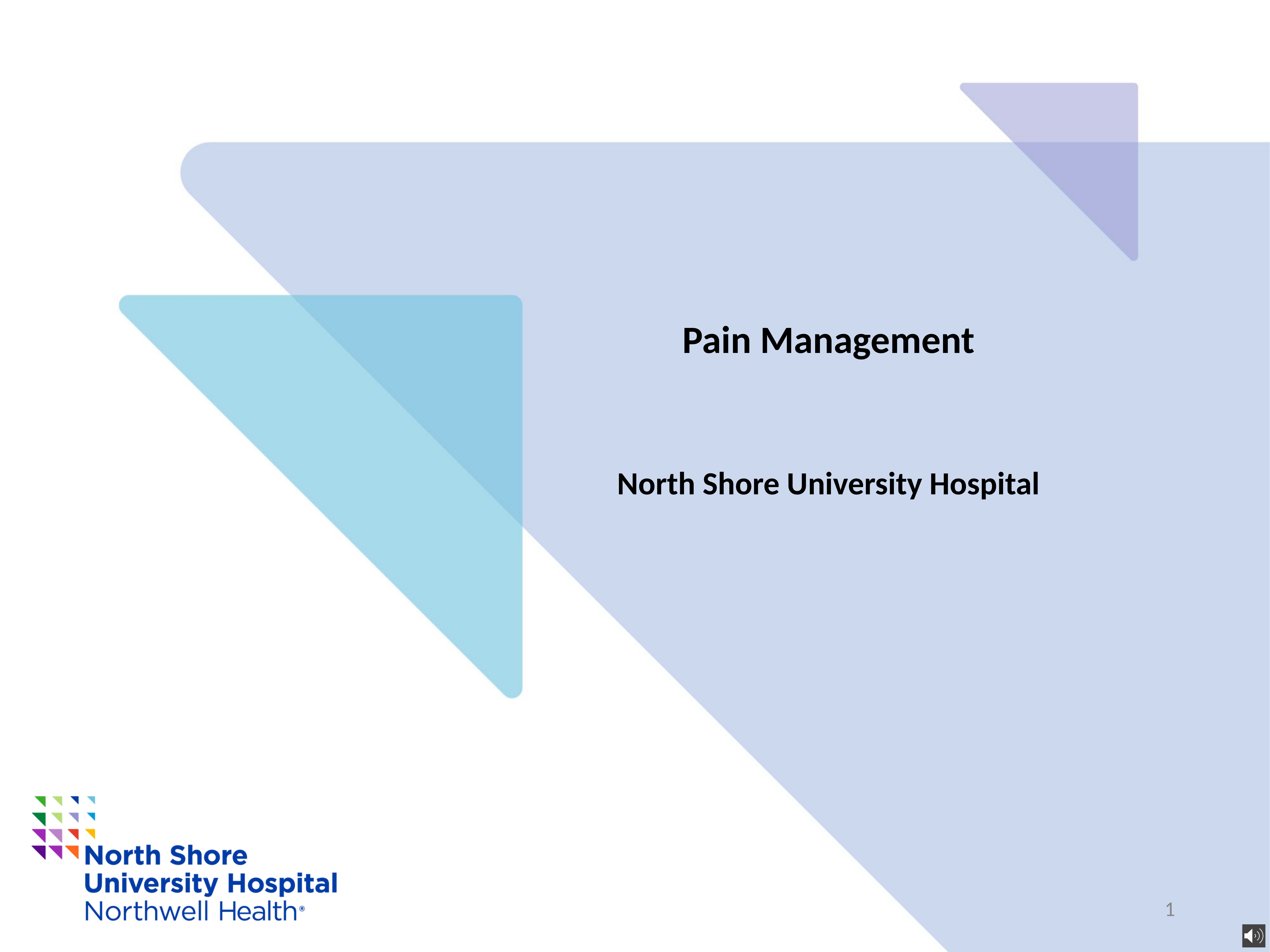

# Pain Management North Shore University Hospital
1

## Slide 2
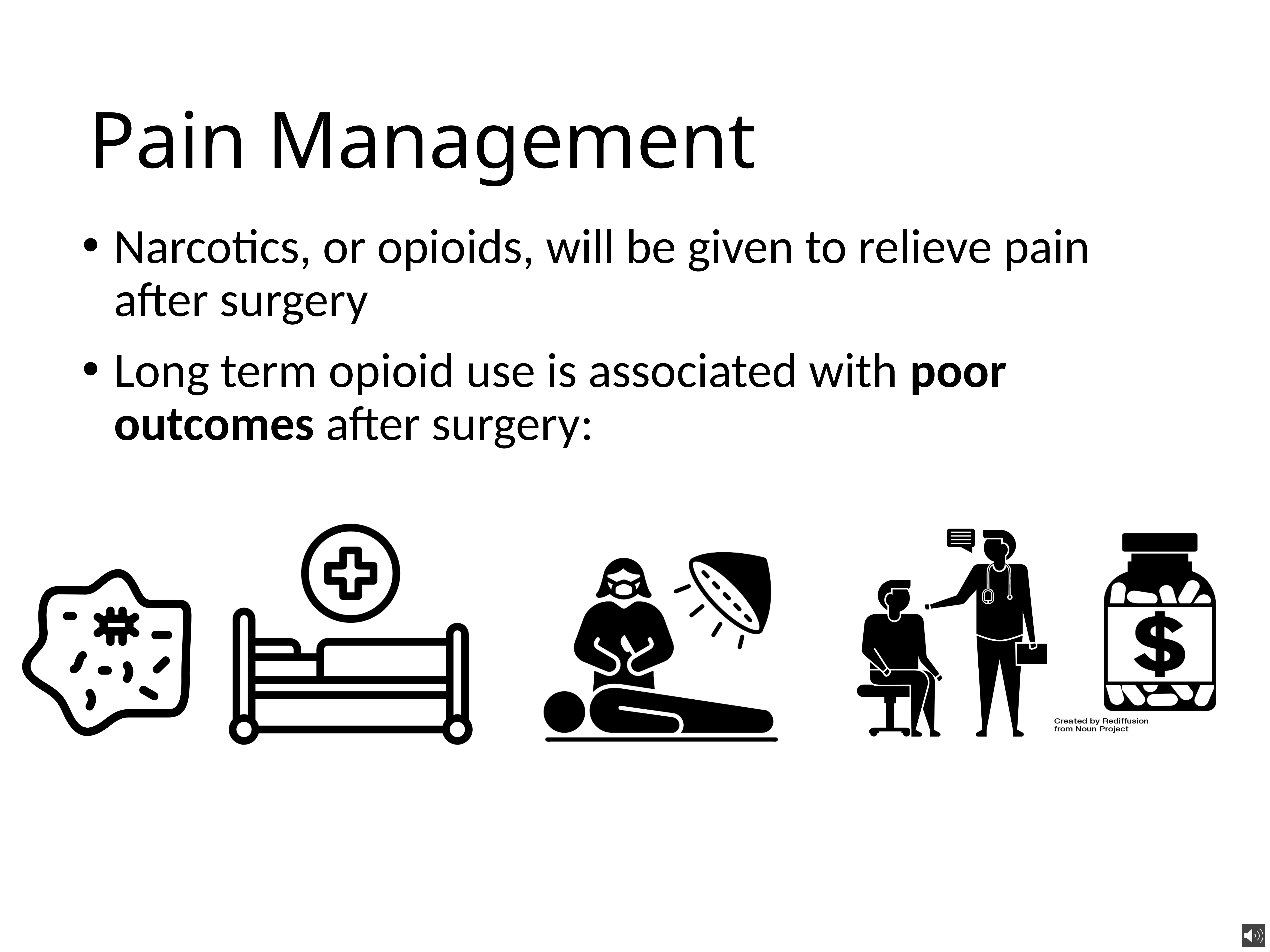

# Pain Management
Narcotics, or opioids, will be given to relieve pain after surgery
Long term opioid use is associated with poor outcomes after surgery:

## Slide 3
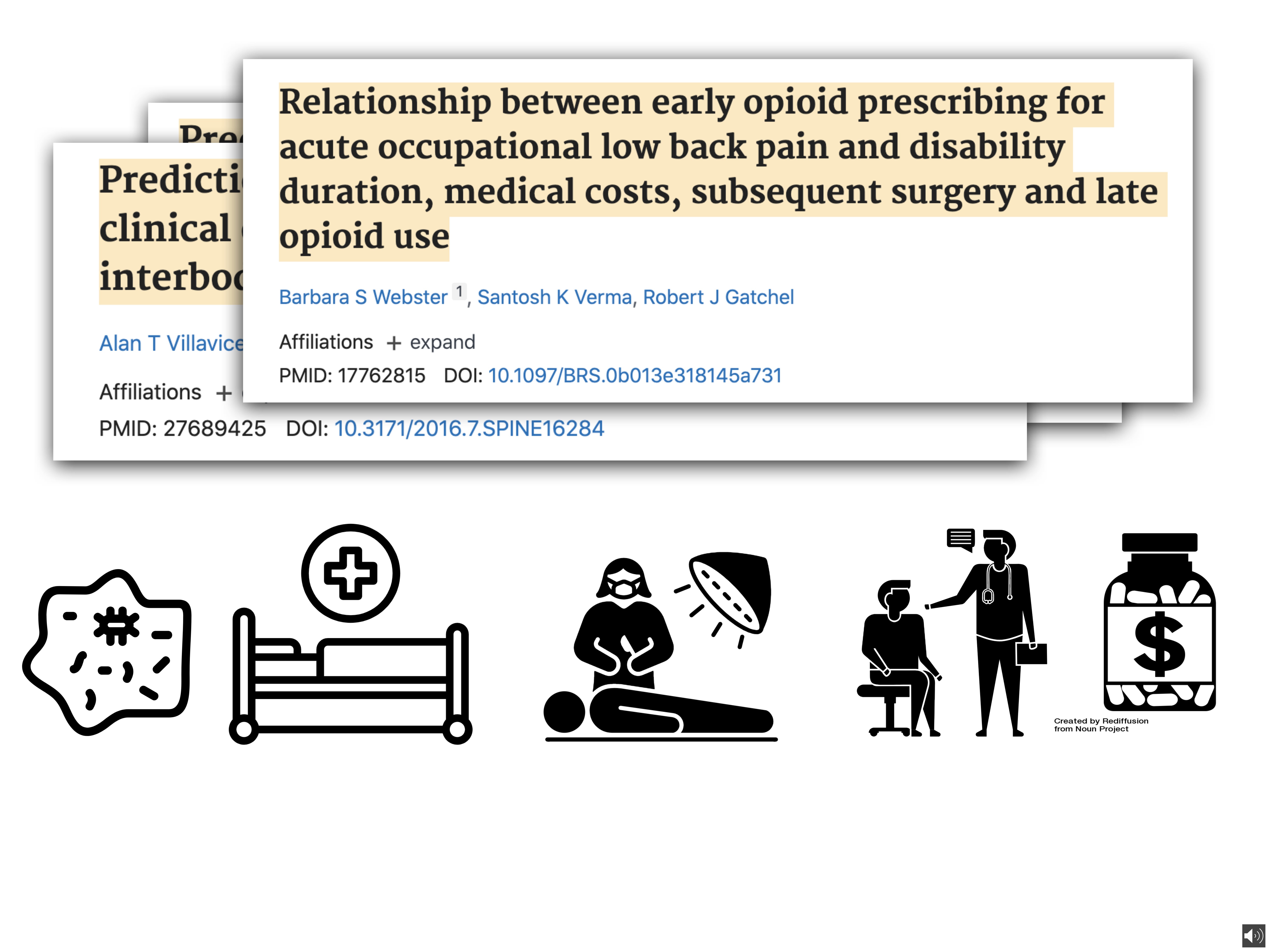

#

## Slide 4
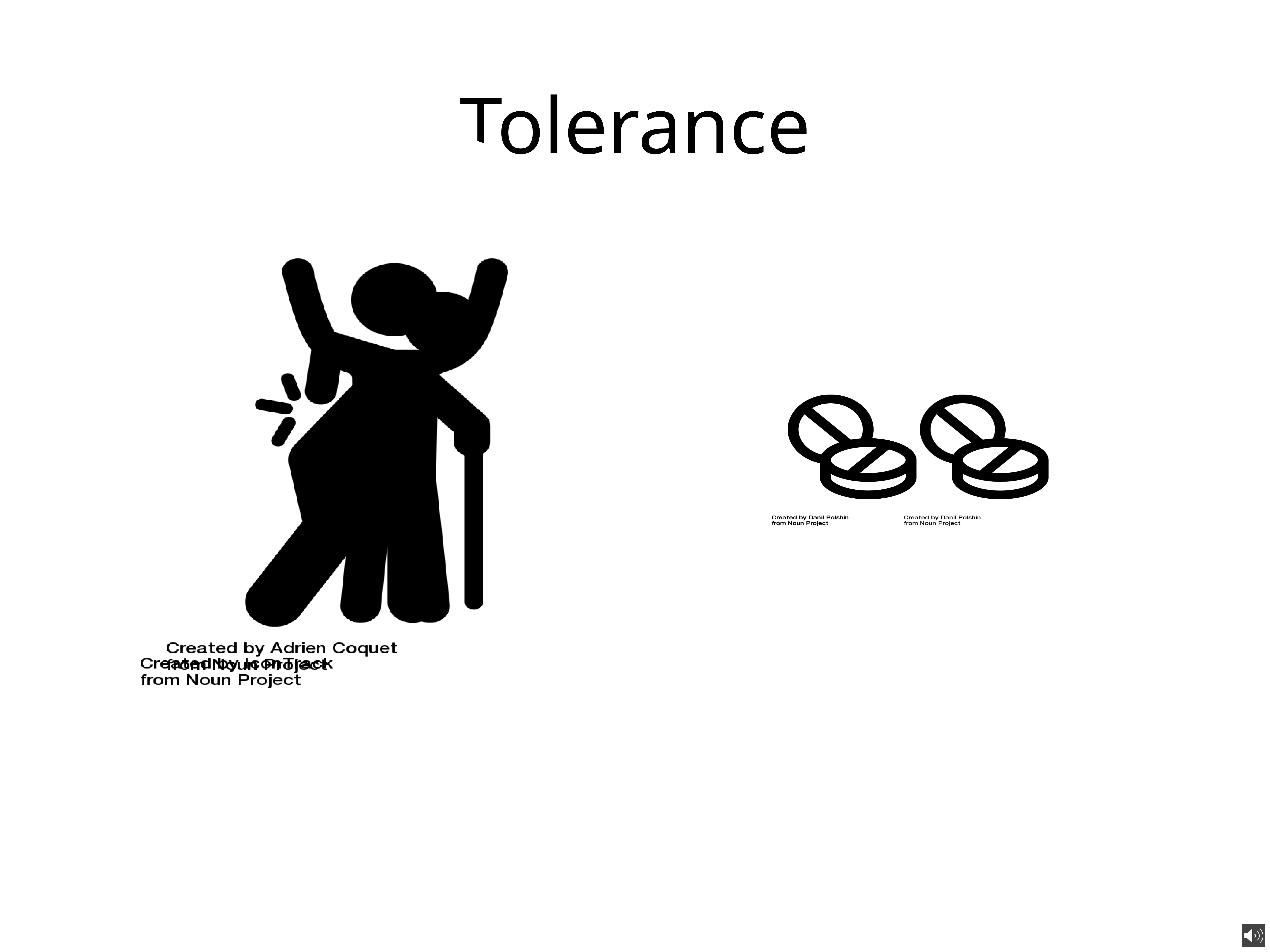

# Tolerance

## Slide 5
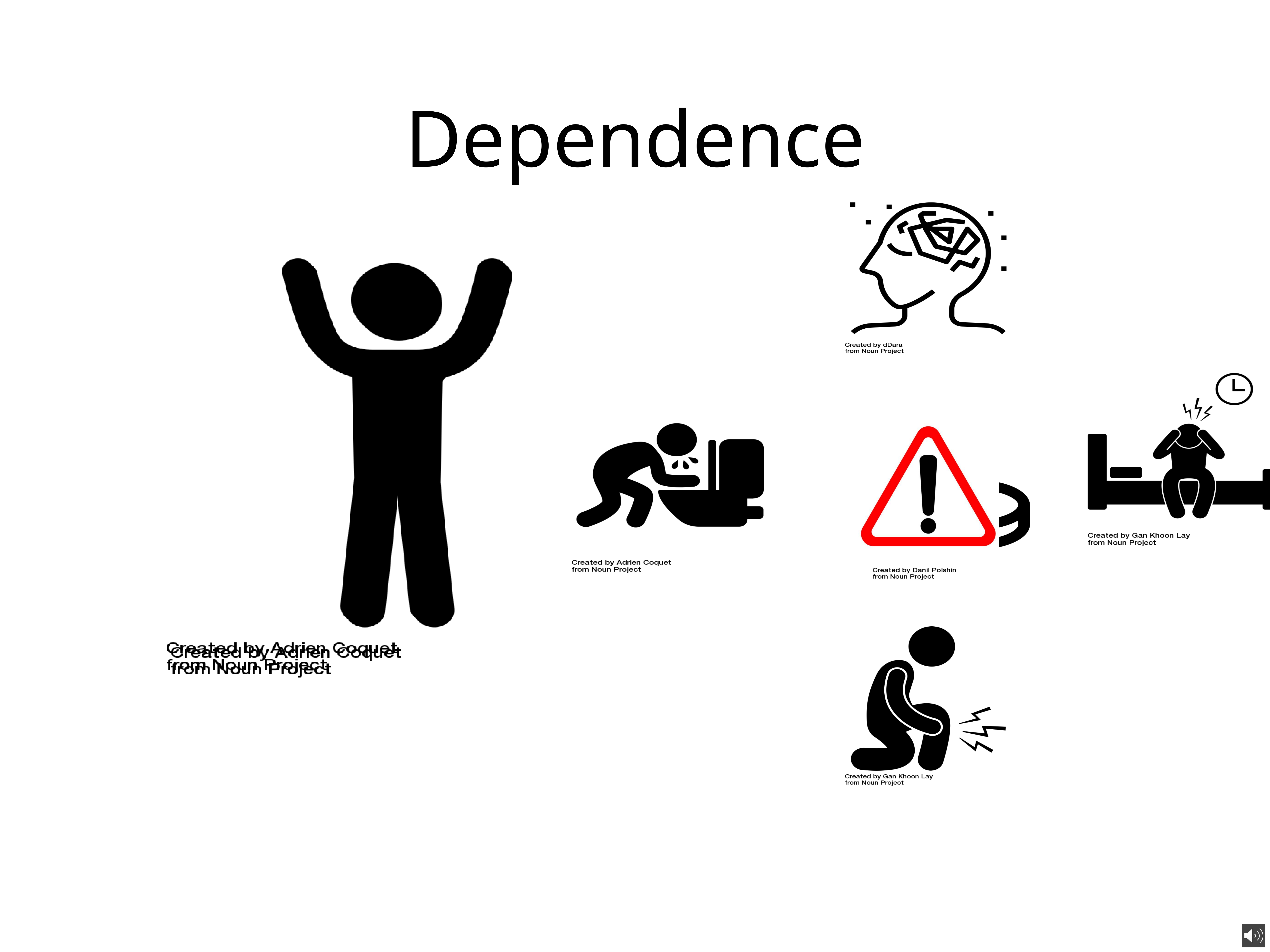

# Dependence

## Slide 6
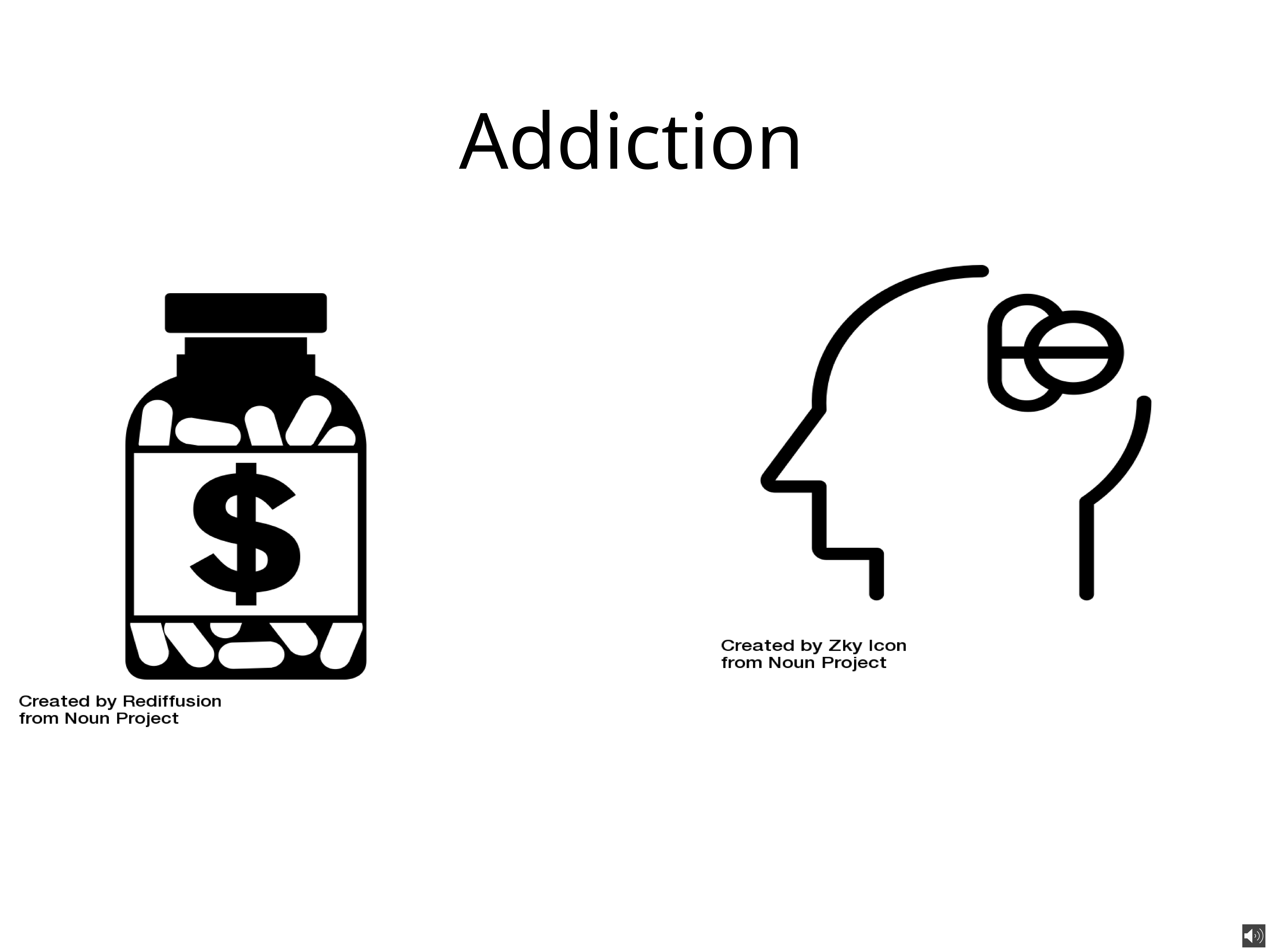

# Addiction

## Slide 7
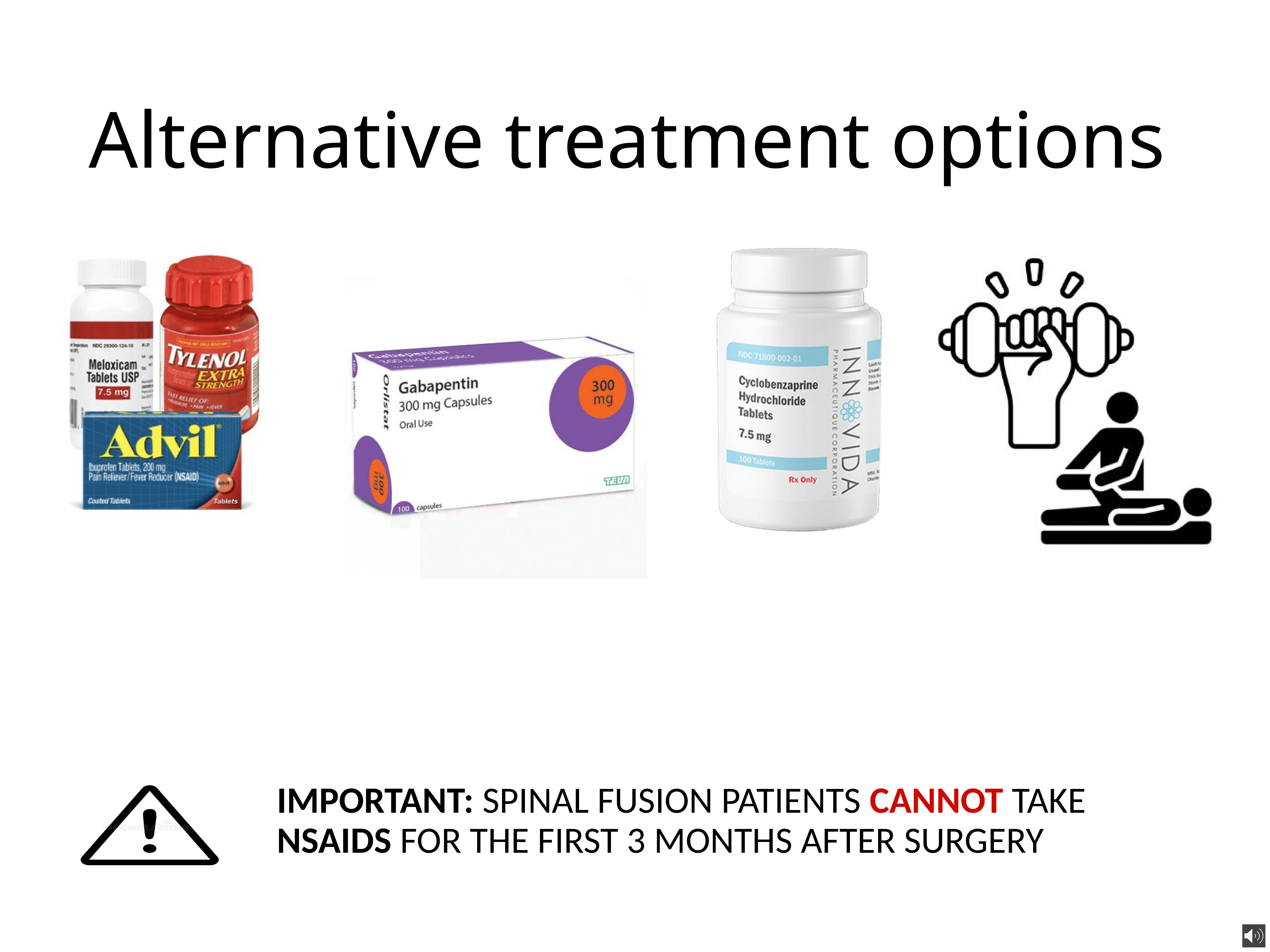

# Alternative treatment options
IMPORTANT: SPINAL FUSION PATIENTS CANNOT TAKE NSAIDS FOR THE FIRST 3 MONTHS AFTER SURGERY

## Slide 8
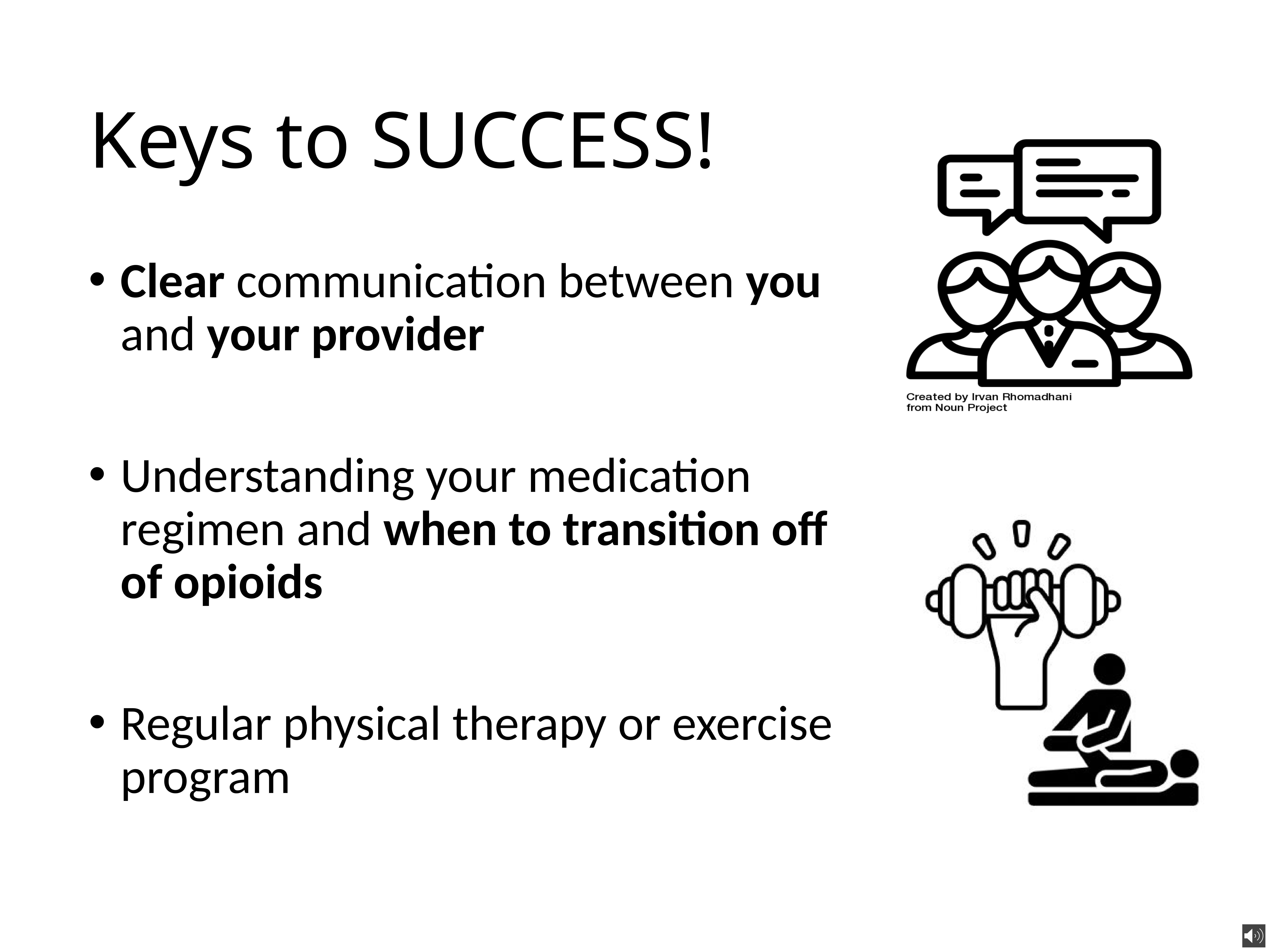

# Keys to SUCCESS!
Clear communication between you and your provider
Understanding your medication regimen and when to transition off of opioids
Regular physical therapy or exercise program

## Slide 9
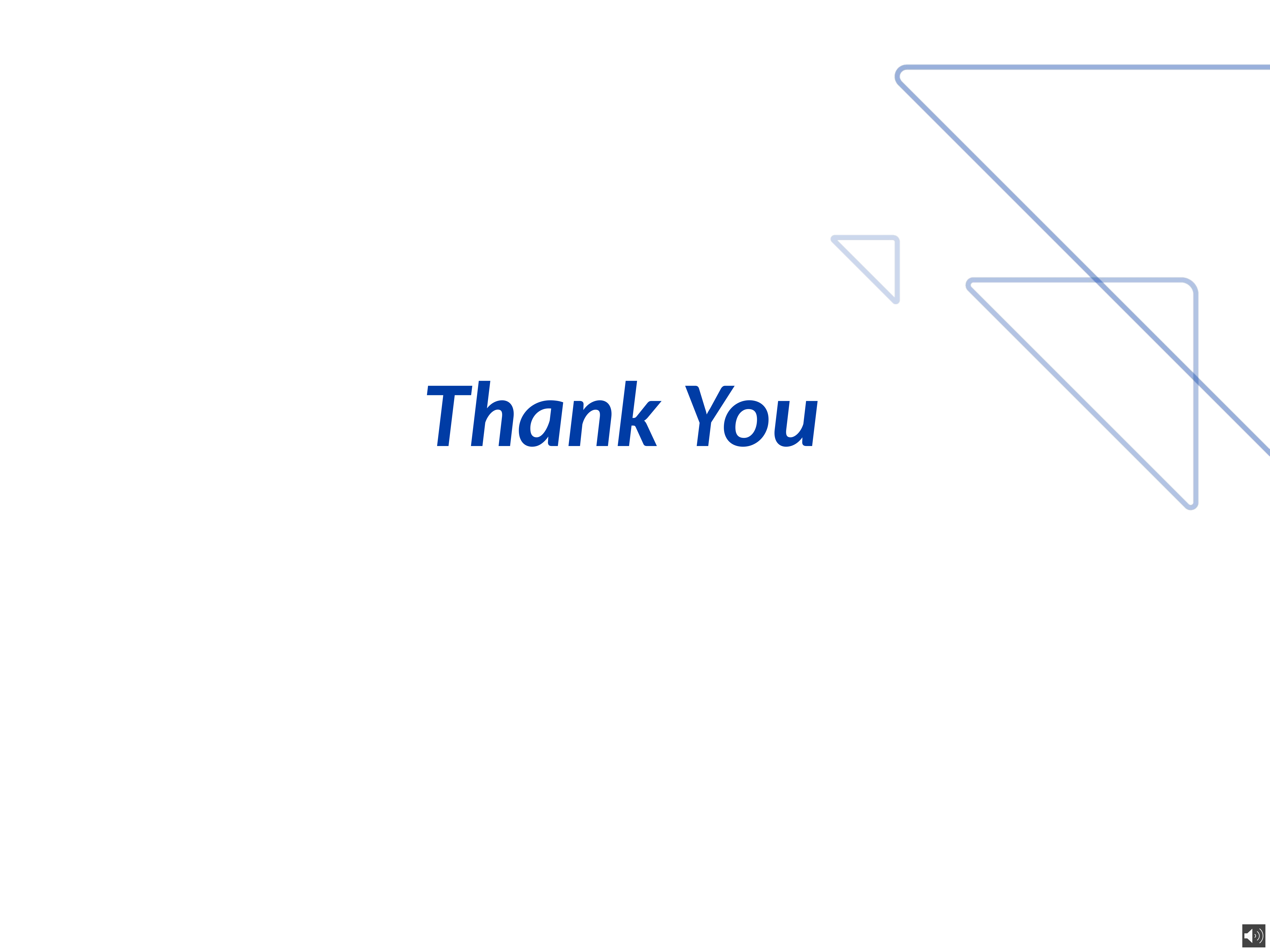

Thank You
