# Supplementary material for: Preoperative Narcotic Education in Spine Surgery: A Retrospective Study
Source: J Clin Med. 2024 Nov 6;13(22):6644. doi: 10.3390/jcm13226644 (PMC11594543; doi:10.3390/jcm13226644)
Supplement: Supplementary file 1 [file jcm-13-06644-s001.zip › Figure S2.pdf]

PAIN MANAGEMENT

You are signed up for surgery at North Shore University Hospital. This guide will go over options provided to you for pain management following your surgery.

1 WHAT ARE OPIOIDS?

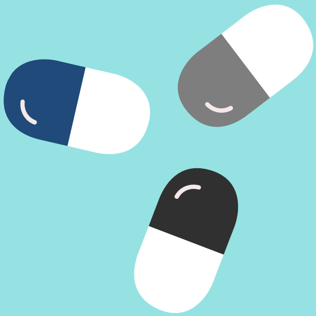

Narcotic medications, also known as opioids, are given after surgery to keep you comfortable while you heal and begin rehabilitation.

2 LONG-TERM EFFECTS OF OPIOID USE

Long term opioid use is associated with poorer outcomes including: infection, longer hospital stay, increased likelihood of revision surgery and additional office visits after surgery, which can result in higher medical costs.

Several studies have shown that nearly half of patients who were on opioid medications before their surgery have one or more these poor outcomes.

3 TOLERANCE

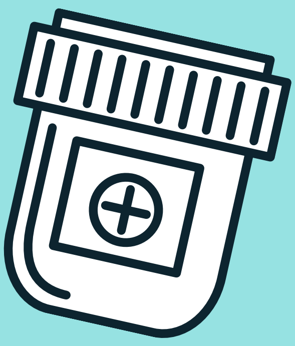

Long term opioid use is also associated with tolerance. Tolerance is when your regular dose no longer works and you need to take a greater dose of your medication to feel the same effects.

4 DEPENDENCE

Dependence is when you begin to feel unpleasant symptoms after stopping your medications. This may cause you to take your medication just to feel “normal”. Withdrawal symptoms include: agitation or anxiety, insomnia, muscle weakness or cramping, and nausea or vomiting.

5 ADDICTION

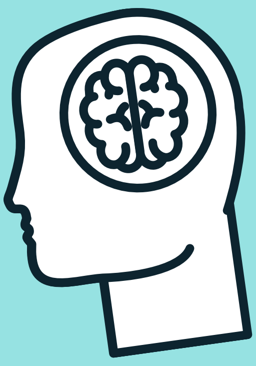

Addiction may cause someone to compulsively use their medications despite having unpleasant side effects. Higher rates of addiction occur with extended, high-dose opioid use.

6 ALTERNATIVES TO OPIOIDS

There are plenty of non-narcotic options that can be helpful in relieving your pain including anti-inflammatories or other pain relievers, neuropathic agents, and muscle relaxants. These medications can be combined with physical therapy for the best recovery.

IMPORTANT: PTS WHO WILL UNDERGO SPINAL FUSION *CANNOT* **TAKE NSAIDS** FOR THE *FIRST 3 MONTHS* AFTER SURGERY AS THIS WILL INHIBIT BONE GROWTH.

7 REMEMBER!

Talk to your doctor about the medications you will be taking after surgery, and when to transition off of opioids to alternative pain relief. Be sure to attend physical therapy regularly for guided rehabilitation.

WE WISH YOU A SPEEDY RECOVERY
